# Snake Cathelicidin NA-CATH and Smaller Helical Antimicrobial Peptides Are Effective against *Burkholderia thailandensis*

**DOI:** 10.1371/journal.pntd.0003862

**Published:** 2015-07-21

**Authors:** Ryan J. Blower, Stephanie M. Barksdale, Monique L. van Hoek

**Affiliations:** 1 George Mason University, School of Systems Biology, Manassas, Virginia, United States of America; 2 George Mason University, National Center for Biodefense and Infectious Diseases, Manassas, Virginia, United States of America; Liverpool School of Tropical Medicine, UNITED KINGDOM

## Abstract

*Burkholderia thailandensis* is a Gram-negative soil bacterium used as a model organism for *B*. *pseudomallei*, the causative agent of melioidosis and an organism classified category B priority pathogen and a Tier 1 select agent for its potential use as a biological weapon. *Burkholderia* species are reportedly “highly resistant” to antimicrobial agents, including cyclic peptide antibiotics, due to multiple resistance systems, a hypothesis we decided to test using antimicrobial (host defense) peptides. In this study, a number of cationic antimicrobial peptides (CAMPs) were tested *in vitro* against *B*. *thailandensis* for both antimicrobial activity and inhibition of biofilm formation. Here, we report that the Chinese cobra (*Naja atra*) cathelicidin NA-CATH was significantly antimicrobial against *B*. *thailandensis*. Additional cathelicidins, including the human cathelicidin LL-37, a sheep cathelicidin SMAP-29, and some smaller ATRA peptide derivatives of NA-CATH were also effective. The D-enantiomer of one small peptide (ATRA-1A) was found to be antimicrobial as well, with EC50 in the range of the L-enantiomer. Our results also demonstrate that human alpha-defensins (HNP-1 & -2) and a short beta-defensin-derived peptide (Peptide 4 of hBD-3) were not bactericidal against *B*. *thailandensis*. We also found that the cathelicidin peptides, including LL-37, NA-CATH, and SMAP-29, possessed significant ability to prevent biofilm formation of *B*. *thailandensis*. Additionally, we show that LL-37 and its D-enantiomer D-LL-37 can disperse pre-formed biofilms. These results demonstrate that although *B*. *thailandensis* is highly resistant to many antibiotics, cyclic peptide antibiotics such as polymyxin B, and defensing peptides, some antimicrobial peptides including the elapid snake cathelicidin NA-CATH exert significant antimicrobial and antibiofilm activity towards *B*. *thailandensis*.

## Author Summary

*Burkholderia* species such as *B*. *pseudomallei*, which causes melioidosis, and the model organism *B*. *thailandensis* are extremely resistant to antibiotics, including cyclic peptide antibiotics such as polymyxin B. Treatment for *Burkholderia* infections is impeded by this resistance, and new approaches are needed. We hypothesized that the cathelicidin NA-CATH from the Chinese cobra, *Naja atra*, and smaller derivative peptides (ATRA peptides) may have antimicrobial activity against *Burkholderia*. We therefore tested the bactericidal effects of the cathelicidin and its derivative peptides. We also wanted to determine whether the antimicrobial peptides exert anti-biofilm activity, although the role of biofilm as a critical virulence factor of *Burkholderia* has not yet been established. We found that the peptide ATRA-1A, as well as the stereo-isomer D-ATRA-1A, were able to kill *B*. *thailandensis*, and the full-length snake cathelicidin NA-CATH was able to both kill *B*. *thailandensis* and inhibit its biofilm formation, unlike the human-alpha defensin peptides HNP-1 and HNP-2, and the small peptide derived from hBD3. These results show that the NA-CATH antimicrobial peptide possess bactericidal and anti-biofilm activity against *B*. *thailandensis*, and suggest that these compounds should be tested for their effect against the more virulent strains of *Burkholderia*.

## Introduction

*Burkholderia pseudomallei* is a Gram-negative soil bacterium which acts as a facultative intracellular pathogen that can infect both humans and animals, causing melioidosis. Melioidosis is endemic to Southeast Asia and Northern Australia, where the mortality rates are 50% and 19% respectively [1–3]. In addition, *B*. *pseudomallei* is of interest because it is considered a class B priority pathogen and a Tier 1 Select Agent and has potential for aerosol delivery. In this study, *Burkholderia thailandensis* is used as a model for *B*. *pseudomallei* [4]. *B*. *thailandensis* is a BSL-2 organism closely related to *B*. *pseudomallei* with an LD50 in mice 1000-fold higher than that of *B*. *pseudomallei*, making it an easier and safer model organism with which to work [5]. *B*. *thailandensis* has been successfully demonstrated to be a useful BSL-2 surrogate for *B*. *pseudomallei* [4,6–8] for both *in vitro* and *in vivo* experiments. Thus, *B*. *thailandensis* may be a good model in which to study the molecular actions of full length cathelicidins such as LL37 both as antibacterial and antibiofilm peptides against *Burkholderia* strains.

In *B*. *pseudomallei* and *B*. *thailandensis* the significant resistance towards several categories of antibiotics, including chloramphenicol, quinolones, tetracyclines, and trimethoprim, is mediated by the overexpression of efflux pumps [9,10]. *B*. *pseudomallei* and *B*. *thailandensis* are typically grown in the laboratory in the presence of >100 mg/ml polymyxin B [11]; such ready growth indicates their high level of resistance to cyclic peptide antibiotics. In fact, the genus *Burkholderia* is said to have “extreme antimicrobial peptide and polymyxin B resistance” [12]. Therefore, the discovery of novel therapeutic alternatives is urgently required.

We have previously studied the cathelicidin peptide from the elapid snake *Naja atra* and designed smaller peptide derivatives called ATRA peptides; we reported that these peptides were highly active against both Gram-positive and Gram-negative bacteria, such as Gram-positive *Staphylococcus* aureus and Gram-negative *Pseudomonas aeruginosa* [13–16]. We were very interested to know whether *B*. *thailandensis* would be susceptible to other antimicrobial peptides, and particularly to the very effective cathelicidin peptide (NA-CATH) and smaller peptide derivatives from elapid snakes that we had been studying.

Cationic antimicrobial peptides (CAMPs) are produced as part of the innate immune system by higher-order organisms. These peptides are also referred to as host-defense peptides (HDPs). CAMPs are low-molecular-weight, cationic, and often amphipathic peptides, and their overall positive charge enables association with the negatively charged bacterial outer membrane [17]. In this study, we tested two types of CAMPs: the cathelicidin type and the defensin type.

It has been previously reported that *Burkholderia* species, specifically *B*. *cepacia*, are very resistant to beta-defensins, a category of defensin CAMPs [18]. Defensins function by replacing Ca^2+^ and Mg^2+^ ions in the bacterial membrane, disrupting membrane stability and leading to loss of electric potential and eventual cell lysis [19,20]. Defensins are important to consider because they are found in human skin under inflammatory conditions [21] and could potentially play a role during a wound infection by *B*. *pseudomallei*. In this study, we tested the antibacterial activity of two alpha-defensins and a small peptide from beta-defensin against *B*. *thailandensis*.

Cathelicidins are a class of antimicrobial peptide characterized by a highly conserved cathelin domain [22] and a sequence-variable active cathelicidin domain. The majority of cathelicidin peptides form amphipathic alpha helices when in contact with a membrane, and these helices are believed to play a crucial role in their function [23,24]. Recently a cathelicidin, designated NA-CATH, has been discovered in *Naja atra*, the Chinese cobra [25], an elapid snake found in Southeast Asia [26]. This cathelicidin contains an imperfect repeated 11-amino-acid motif named the ATRA motif (Table 1) [13]. The first repeat is called ATRA-1 and the second repeat ATRA-2 [13]. A derivative, ATRA-1A, was created by replacing the 3rd residue of ATRA-1 with an alanine [13]. In previous work we demonstrated that the full-length cathelicidin (NA-CATH) and peptides based on the first repeat (ATRA-1 and ATRA-1A) were effective broad-spectrum antimicrobial agents against *Francisella novicida*, *Aggregatibacter actinomycetemcomitans*, *Pseudomonas aeruginosa*, and *Staphylococcus aureus* [13–16]. Therefore, the *Naja atra* cathelicidin NA-CATH and its ATRA derivatives were chosen for studies against *Burkholderia* species [10]. It is of note that we previously demonstrated that the cathelicidins LL-37, D-LL-37, NA-CATH, and NA-CATH derivatives cause no hemolysis at the antimicrobial concentrations used in our study [13,14]. This leads us to suggest that these peptides may be very useful as a potential new therapeutic approach, perhaps in a topical application, by virtue of their demonstrated antimicrobial action and minimal host-cell cytotoxicity, with the D-peptides having the added advantage of less susceptibility to protease digestion.

**Table 1:** Sequences of antimicrobial peptides and their respective net charges.

Peptide	Sequence	Charge
LL-37 [16]	LLGDFFRKSKEKIGKEFKRIVQRIKDFLRNLVPRTES	+6
SMAP-29	RGLRRLGRKIAHGVKKYGPTVLRIIRIAG	+9
NA-CATH [13]	KRFKKFFKKLKNSVKKRAKKFFKKPKVIGVTFPF	+15
ATRA-1 [13]	KRFKKFFKKLK	+8
ATRA-1A [13]	KRAKKFFKKLK	+8
ATRA-2	KRAKKFFKKPK	+8
Peptide 4 of hBD-3 [42]	RGRKSSRRKK	+7
HNP-1 [57]	ACYCRIPACIAGERRYGTCIYQGRLWAFCC	+3
HNP-2 [57]	CYCRIPACIAGERRYGTCIYQGRLWAFCC	+3
Scrambled LL-37 [14,15]	GLKLRFEFSKIKGEFLKTPEVRFRDIKLKDNRISVQR	+6

## Methods

### Bacterial cells

*B*. *thailandensis* (E264) was obtained from the American Type Culture Collection (Manassas, VA), ATCC 700388, and grown in nutrient broth overnight in a shaking incubator at 37°C. Cultures of *B*. *thailandensis* were grown up and the stocks were aliquotted, frozen in 20% glycerol, and stored at -80°C. Cultures were enumerated by serial dilution on nutrient agar.

### Antimicrobial assays

The antimicrobial activity of various antimicrobial peptides against *B*. *thailandensis* was determined as previously described [16]. Briefly, in a sterile 96-well plate, 1x10^5^ CFU per well of bacteria were incubated with serial dilutions of antibiotic (control) and peptide in 10 mM phosphate buffer (3 h, 37°C). Bacterial survival was then determined by serial dilution at each peptide concentration in sterile PBS. Dilutions were plated in triplicate on nutrient agar and incubated at 37°C for 24 h; colonies were then counted to determine survival. Bacterial survival was calculated by the ratio of the number of colonies on each experimental plate to the average number of colonies in the control plates lacking any antimicrobial peptide.

The antimicrobial peptide concentration required to kill 50% of *B*. *thailandensis* (EC50) was determined by graphing percent survival versus log of peptide concentration (log μg/ml). Data were plotted using GraphPad Prism 5 (GraphPad Software Inc., San Diego, CA, USA). Survival was determined as the ratio of colonies from experimental plates relative to the average number of colonies from plates lacking peptide. EC50 was determined by fitting the data to a standard sigmoidal dose-response curve. Each experiment was performed three times with three replicates per experiment for n = 9. Error is reported as 95% confidence intervals (CI) for each antimicrobial peptide.

### Biofilm inhibition assays

Biofilm was grown and measured as previously described [27–29]. Modified Vogel and Bonner’s medium (MVBM) [30] was inoculated from an overnight culture of *B*. *thailandensis* and allowed to incubate for 18 h in a shaking incubator at 37°C. The optical density at 540 nm (OD_540_) was adjusted to 0.8 OD_540_. Bacterial suspension (100 μl) was added to wells of a sterile tissue-culture-treated 96-well plate along with various concentrations of peptide and fresh MVBM (final volume 200 μl). Wells containing only medium or no peptide served as negative and positive controls, respectively. Plates were then incubated aerobically at 37°C for 3 h. Following aerobic adhesion, supernatant fluid was removed from wells (to remove planktonic bacteria), fresh MVBM/peptide was added to each well (200 μL final volume), and plates were incubated for 21 h at 37°C. After incubation, supernatant was removed and replaced with 200 μL of fresh MVBM/peptide, then incubated at 37°C for an additional 24 h. This is described as a 48 h biofilm. After final incubation, the plate was read at OD_600nm_ to measure bacterial growth, then washed, fixed, and stained with crystal violet as previously described [31]. Each assay was performed in triplicate and the experiment repeated three times for n = 9.

### Biofilm dispersion assays

Biofilm dispersion assay was performed using *B*. *thailandensis* E264 (ATCC 700388) in 100 μL MVBM and was incubated 24h, 37°C. After allowing biofilm to form for 24h, the biofilm was treated with 10 μg peptide or 0 peptide and then incubated at 37°C for an additional 24h. The optical density was measured prior to staining to measure bacterial growth after 48h incubation. Eight wells were used for each peptide (n = 8). Production of biofilm was measured using crystal violet staining as described previously [31].

### Protein ID numbers

Protein ID numbers were obtained from the UniProt protein database. The protein ID number of LL-37, the human cathelicidin, is P49913. The protein number for SMAP-29, the sheep cathelicidin, is P49928. The protein number for NA-CATH, the cathelicidin from the Chinese King Cobra, is B6S2X0.

## Results

### Cathelicidin peptides demonstrate antimicrobial effects against *B*. *thailandensis*

**Fig 1:**
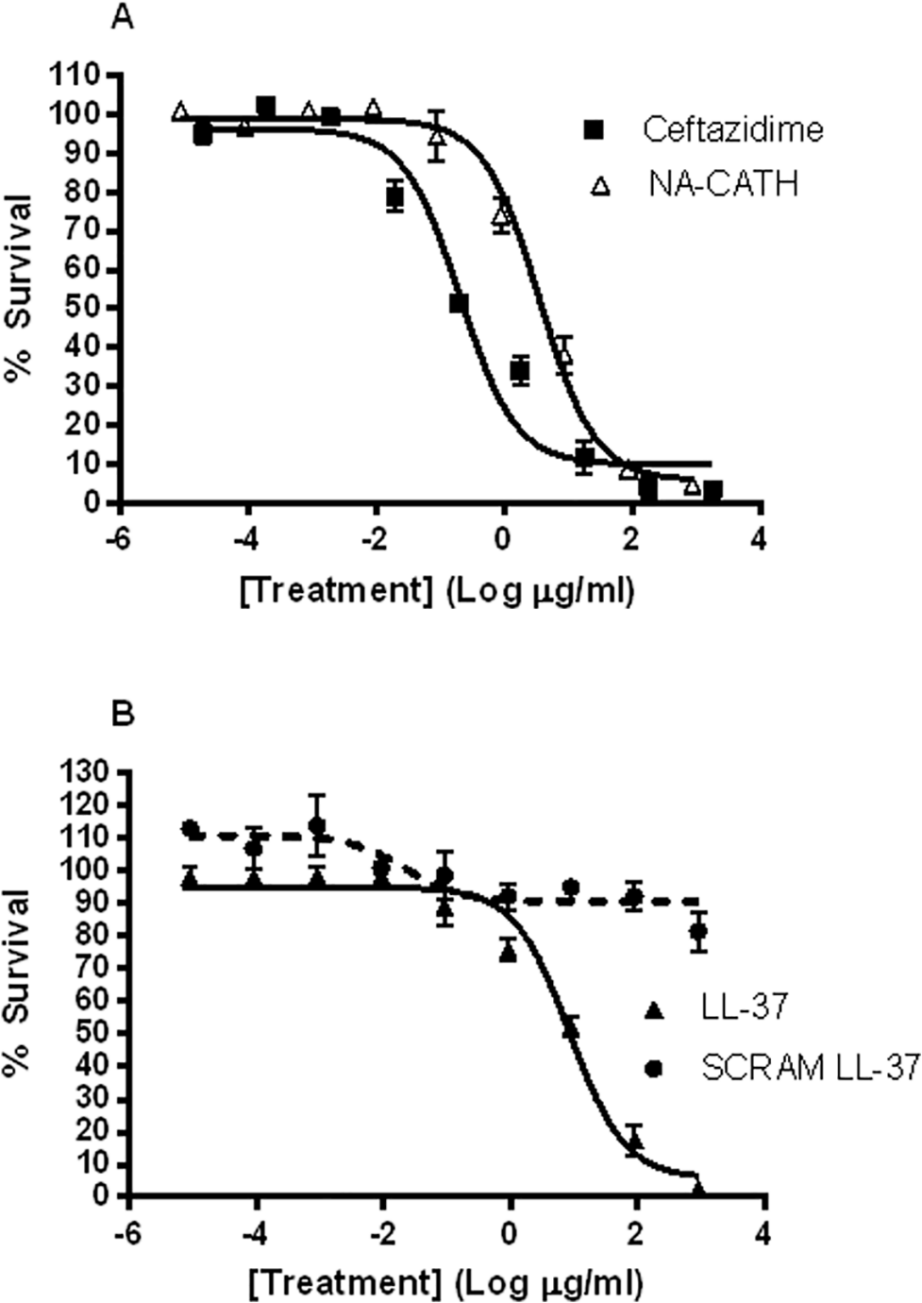
Cathelicidin antimicrobial activity against *B*. *thailandensis*. *B*. *thailandensis* was incubated for 3 h with various peptide concentrations in 10 mM sodium phosphate buffer (pH 7.4), and percent (%) survival was calculated as the ratio of CFUs before and after incubation. **(A)** EC50 for the positive control ceftazidime and for NA-CATH. **(B)** EC50 for LL-37 and SCRAM-LL-37.

In this study, we demonstrated the effectiveness of various snake-derived cathelicidin peptides against *B*. *thailandensis*, including NA-CATH. Control peptides included SMAP-29 and LL-37 (Table 2). Ceftazidime is the first-line antibiotic against *B*. *pseudomallei* [32]. Therefore, it was used as a positive control for observing a bactericidal effect in the antimicrobial plating assay. We determined the first-line antibiotic ceftazidime to have an EC50 value of 0.328 μM (95% CI of 0.20–0.548 μM) (Fig 1).

**Table 2:** Antimicrobial activity and confidence intervals of the peptide panel.

Peptide	Molecular Weight (g/mol)	EC50 (μg/ml)	Confidence Interval (μg/ml)	EC50 (μM)	Confidence Interval (μM)
Ceftazidime	636.6	0.209	0.125–0.349	0.328	0.20–0.548
LL-37	4493.3	8.43	5.37–13.2	1.87	1.20–2.94
SMAP-29	3256	2.05	0.633–6.61	0.628	0.194–2.03
NA-CATH	4175.22	3.66	2.55–5.26	0.877	0.61–1.26
ATRA-1	1497.92	10.4	6.38–17.0	6.94	4.26–11.3
ATRA-1A	1420.84	14.0	9.27–21.1	9.83	6.52–14.8
D-ATRA-1A	1420.84	6.86	4.54–10.33	4.82	3.20–7.27
D-LL-37	4493.3	16.4	9.16–29.4	3.64	2.04–6.53

Scrambled LL-37, ATRA-2, and HNP-2 are not shown as no EC50 could be determined.

We found that the *Naja atra* peptide NA-CATH had EC50 values of 0.877 μM (95% CI of 0.61–1.26 μM) against *B*. *thailandensis*. This compared favorably to the sheep peptide SMAP-29, which was observed to have an EC50 value of 0.628 μM (95% CI of 0.194–2.03 μM). The human cathelicidin LL-37 was also found to have a good antimicrobial effect against *B*. *thailandensis*, with an EC50 value of 1.87 μM (95% CI of 1.20–2.94 μM). Data are presented in μM to reflect the number of peptide molecules, thereby compensating for differing molecular weights. These results are consistent with the published values for the effect of LL-37 against *Burkholderia* [33–35]. These three cathelicidin peptides (NA-CATH, SMAP-29, LL-37) were not statistically different in their anti-*B*. *thailandensis* performance. The activity of LL-37 is similar to that shown for *B*. *pseudomallei* [34]. In previous work, these same cathelicidin peptides were tested against *P*. *aeruginosa*, *S*. *aureus*, and *F*. *novicida* [14–16]. We had expected *Burkholderia* species to have a higher EC50 than those organisms because of their wide range of mechanisms to evade destruction by antibiotics and antimicrobial peptides [10]. Surprisingly, the EC50 results against *B*. *thailandensis* were similar to cathelicidin EC50 values against other Gram-negative bacteria [14–16].

### Small synthetic (ATRA) peptides derived from the *Naja atra* cathelicidin exhibited significant antimicrobial activity

**Fig 2:**
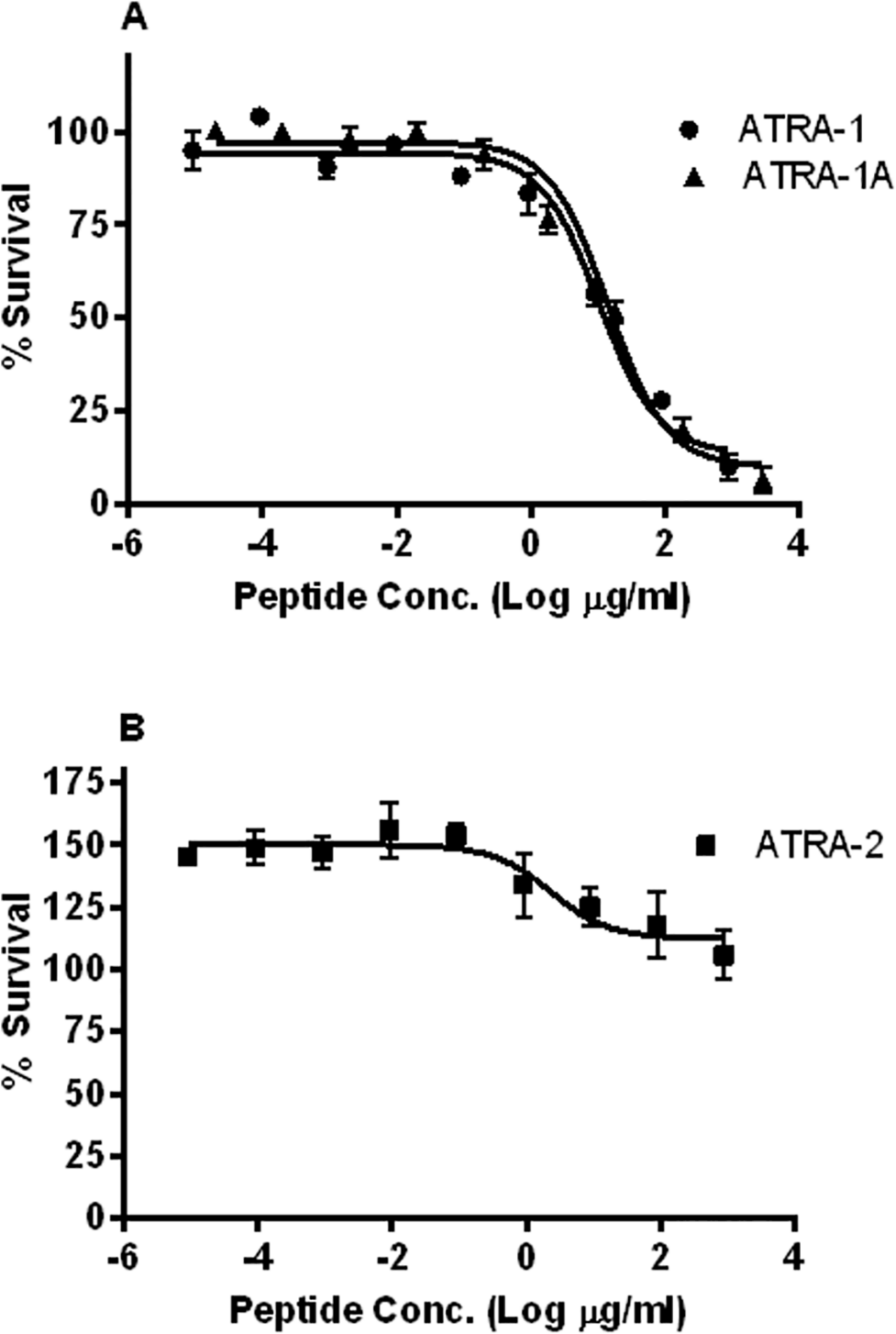
Antimicrobial activity of NA-CATH derivatives against *B thailandensis*. *B*. *thailandensis* was incubated for 3 h with various peptide concentrations in 10 mM sodium phosphate buffer (pH 7.4); percent (%) survival was calculated as the ratio of CFUs before and after incubation. (**A**) EC50 of ATRA-1 and ATRA-1A. (**B**)ATRA-2 did not exhibit antimicrobial activity.

*Naja atra* cathelicidin peptide derivatives were tested for antimicrobial activity against *B*. *thailandensis*. Each imperfect repeat from NA-CATH (ATRA-1 and ATRA-2) was tested, as well as the synthetic peptide ATRA-1A, in which amino acid 3 of ATRA-1 was switched from phenylalanine to alanine [13] [26]. The EC50 of ATRA-1 against *B*. *thailandensis* was determined to be 6.94 μM (95% CI of 4.26–11.3 μM) (Fig 2). EC50 plating assays determined that ATRA-2 was not an effective antimicrobial peptide, correlating with our previous ATRA-2 results with other bacteria [14–16]. This leads to the conclusion that the first imperfect repeat of NA-CATH contributes to most of the observed antimicrobial activity of NA-CATH. We then looked at a synthetic peptide, named ATRA-1A, which contains a single amino acid change at position 3 (F->A). This synthetic peptide exhibited an EC50 of 9.83 μM (95% CI of 6.52–14.8 μM).

### D-amino acid stereoisomer forms of cathelicidin antimicrobial peptides exert antimicrobial activity against *B*. *thailandensis*

In previous work we demonstrated that peptides produced with each amino-acid in the D-form (enantiomer) can be antimicrobial [14,15,36]. In addition, peptides in the D-form are resistant to proteases such as trypsin [14,15,37,38]. Dean et al. demonstrated that while the L-form of the LL-37 peptide is digested by trypsin, the D-form shows no degradation after 1 h trypsin digestion [15]. Thus, we synthesized all-D-enantiomers of LL-37 and ATRA-1A to compare the antimicrobial activity of these protease-resistant enantiomers.

**Fig 3:**
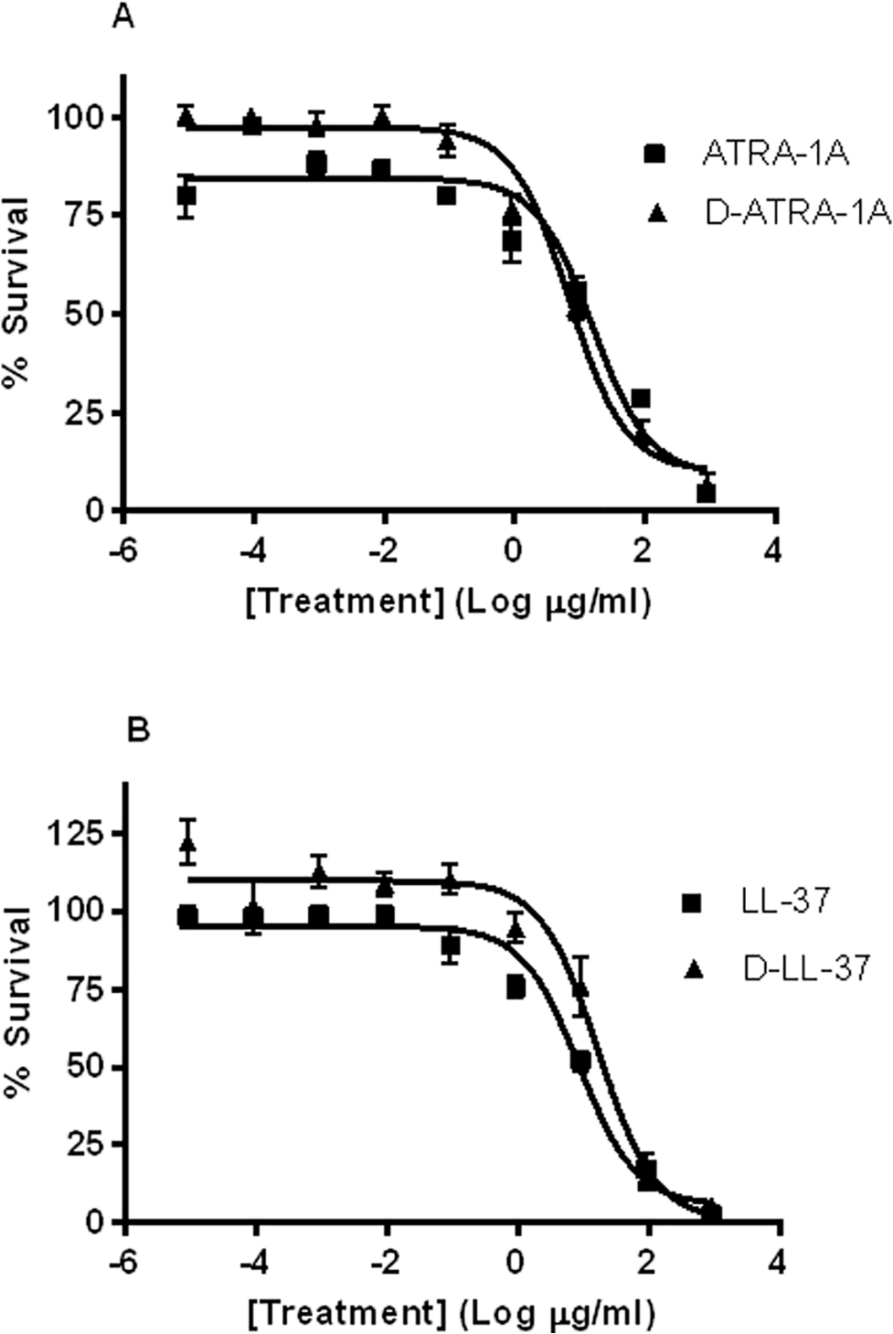
Effect of D-enantiomer on antimicrobial activity. *B*. *thailandensis* was incubated for 3 h with various peptide concentrations in 10 mM sodium phosphate buffer (pH 7.4); percent (%) survival was calculated as the ratio of CFUs before and after incubation. **(A)** EC50 for ATRA-1A and for D-ATRA-1A. **(B)** EC50 for LL-37 and D-LL-37.

We found the antimicrobial effect of the D-enantiomer to be comparable to that of the L-enantiomer for both ATRA-1A and LL-37 (Fig 3). LL-37 had an EC50 value of 1.87 μM (95% CI of 1.20–2.94 μM), while D-LL-37 had a statistically similar EC50 of 3.64 μM (95% CI of 2.04–6.53 μM). D-ATRA-1A had an EC50 value of 4.82 μM (95% CI of 3.20–7.27 μM, as compared to 9.83 μM (95% CI of 6.52–14.8 μM) for ATRA-1A. For both enantiomeric conversions, the 95% confidence intervals of the D-peptide results overlapped those from the normal L version of the peptide. These data demonstrate that converting each peptide to an all-D-enantiomer did not statistically alter its antimicrobial effect.

### Human alpha-and beta-defensins do not exert antimicrobial effects against *B*. *thailandensis*

**Fig 4:**
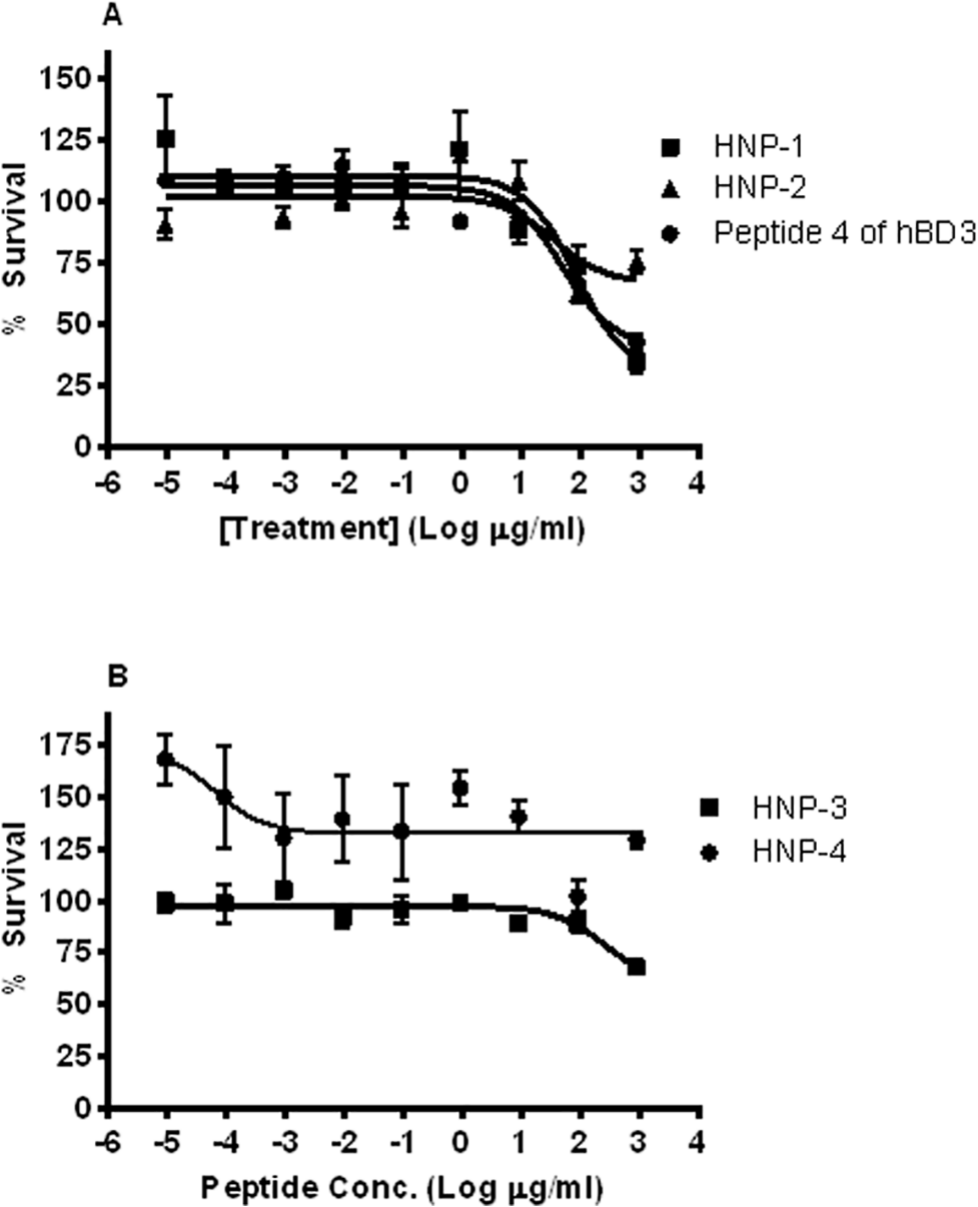
Antimicrobial activity of a panel of defensins against *B*. *thailandensis*. *B*. *thailandensis* was incubated for 3 h with various peptide concentrations in 10 mM sodium phosphate buffer (pH 7.4); percent (%) survival was calculated as the ratio of CFUs before and after incubation. EC50 for these peptides could not be calculated because the peptide was ineffective. (**A**) HNP-1, HNP-2, peptide 4 of hBD3 are depicted. (**B**) HNP-3 and HNP-4 are shown.

We examined a second category of CAMPs, the defensins, for anti-*Burkholderia* activity. Under conditions of *B*. *pseudomallei* respiratory infection in mice, neutrophil granules were observed to be the predominant cell type seen in association with *B*. *pseudomallei* infection [39]. Neutrophil granules are known to be a significant source of cathelicidins and human neutrophil peptides (alpha-defensins) [40]. Therefore, to further explore the effect of defensins upon *B*. *thailandensis*, human alpha defensin-1 (aka human neutrophil peptide 1, HNP-1) and human alpha defensin-2 (HNP-2) were chosen as candidates to test for antimicrobial killing against *B*. *thailandensis* (Fig 4). For HNP-1, at the highest concentration tested (1000 μg/ml peptide), only 65% killing could be achieved for *B*. *thailandensis*, suggesting that this is a highly ineffective peptide. HNP2 was even less effective than HNP1 in killing *B*. *thailandensis* at every concentration tested.

Sahly et al. demonstrated that the LD50 of human beta-defensin-3 (hBD-3) against multiple *Burkholderia* species was >100 μg/ml [18]. However, other reports demonstrated that regions of cationic peptides in the C-terminus of hBD-3 possessed antimicrobial activity against *E*. *coli* and *P*. *aeruginosa* [41,42]. Based on our previous work, we tested a small fragment (Peptide 4) of human beta-defensin 3 (hBD-3), which was previously shown to have significant activity against another Gram-negative bacterium, *E*. *coli* [42]. Incubation of *B*. *thailandensis* with Peptide 4 of hBD-3 at the highest concentration tested (1000 μg/ml) resulted in only 65% killing for *B*. *thailandensis*, suggesting that this is a highly ineffective peptide. These findings confirmed published reports [18,43] that the beta-defensin CAMPs are ineffective against *Burkholderia* species, and demonstrated that the alpha-defensins are also ineffective against *B*. *thailandensis*.

### Cathelicidin antimicrobial peptides inhibit biofilm formation

**Fig 5:**
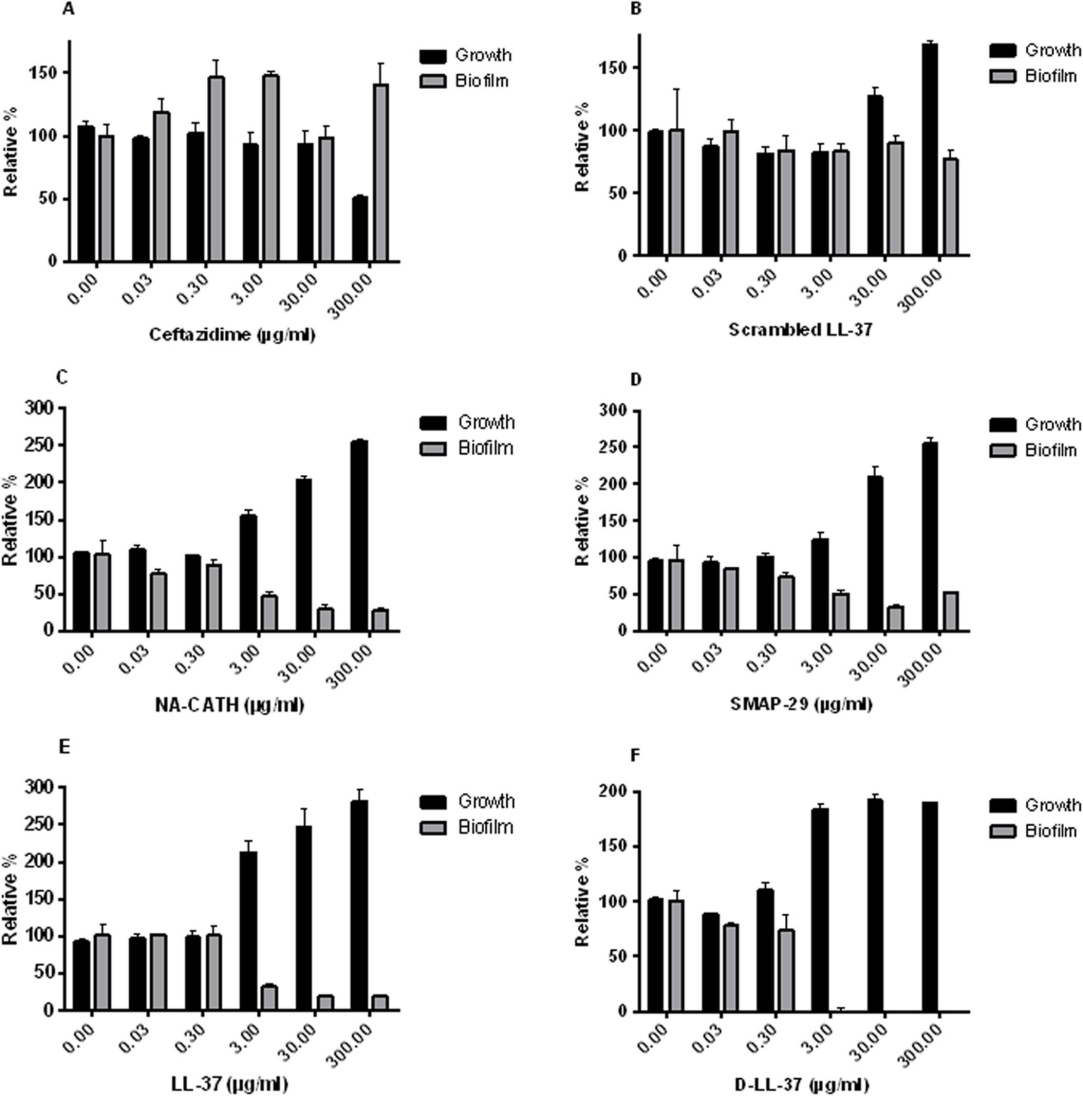
Biofilm activity of cathelicidins against *B*. *thailandensis*. Inhibition of biofilm is demonstrated for the cathelicidins, **C.** NA-CATH, **D.** SMAP-29, **E.** LL-37, and **F.** D-LL-37, while controls **A.** the antibiotic ceftazidime, and **B.** scrambled LL-37 did not show biofilm inhibition. Growth (absorbance at 600 nm) is shown by black bars; growth with no peptide was set to 100%. Biofilm (gray bars) was detected on a polystyrene 96-well plate at 37°C after 48 h of growth in MVBM and detected as absorbance of crystal violet stain (590 nm). Each experiment is representative of 3 individual experiments.

As *B*. *pseudomallei* has been reported to form biofilm [44,45], we sought to demonstrate the ability of various cathelicidins to inhibit biofilm formation in the model organism *B*. *thailandensis*. In addition, we and others have demonstrated that the cathelicidin LL-37 inhibits biofilm formation in *P*. *aeruginosa* [15,46], an important gram-negative pathogen. Therefore, a panel of cathelicidins was tested in biofilm inhibition assays. We first had to establish conditions under which the biofilm of *B*. *thailandensis* could be reliably formed and measured. To do this, we grew the bacteria in modified Vogel-Bonner medium (MVBM) [30] overnight and then adjusted OD_540_ to 0.8. Following growth and measurement, a biofilm inhibition assay was performed. This assay incubates the test compound with the bacteria and determines if the compound can inhibit biofilm formation. The growth of bacteria is also measured to control for bactericidal effects of the test compound although bactericidal effects are reduced due to the high salt concentration of the media used in these assays. We demonstrated that the antibiotic ceftazidime did not inhibit biofilm production but simply killed the *B*. *thailandensis* (Fig 5), as we would expect. Interestingly, the cathelicidins LL-37, SMAP-29, and NA-CATH all showed at least 50% biofilm inhibition at peptide concentrations at or above 3 μg/ml. The negative control peptide, which was a scrambled LL-37 (same amino acid composition and net charge, different sequence of amino acids), did not inhibit biofilm, as we previously reported [14].

In addition, the D-enantiomers of the peptides were tested for their effect on biofilm inhibition. D-LL-37 produced results similar to those of its L-enantiomer. Both inhibited at least 50% of biofilm at concentrations as low as 3 μg/ml. Thus both the L- and D- form of LL-37 exhibit anti-biofilm activity against *B*. *thailandensis*. When the ATRA-1A enantiomers were compared, the results differed slightly (S1 Fig). ATRA-1A did not inhibit biofilm formation, whereas D-ATRA-1A did slightly, but only at the highest concentration of peptide tested (300 μg/ml). At these levels, this is unlikely to be a significant activity of the D-ATRA-1A peptide. ATRA-1 and ATRA-2, the imperfect repeats from NA-CATH, were also tested for biofilm inhibition and did not inhibit biofilm formation (S1 Fig). Thus, the full-length cathelicidin peptides including the snake cathelicidin NA-CATH were able to inhibit *B*. *thailandensis* biofilm formation.

### LL-37 and its D-enantiomer D-LL-37 can disperse pre-formed biofilms

**Fig 6:**
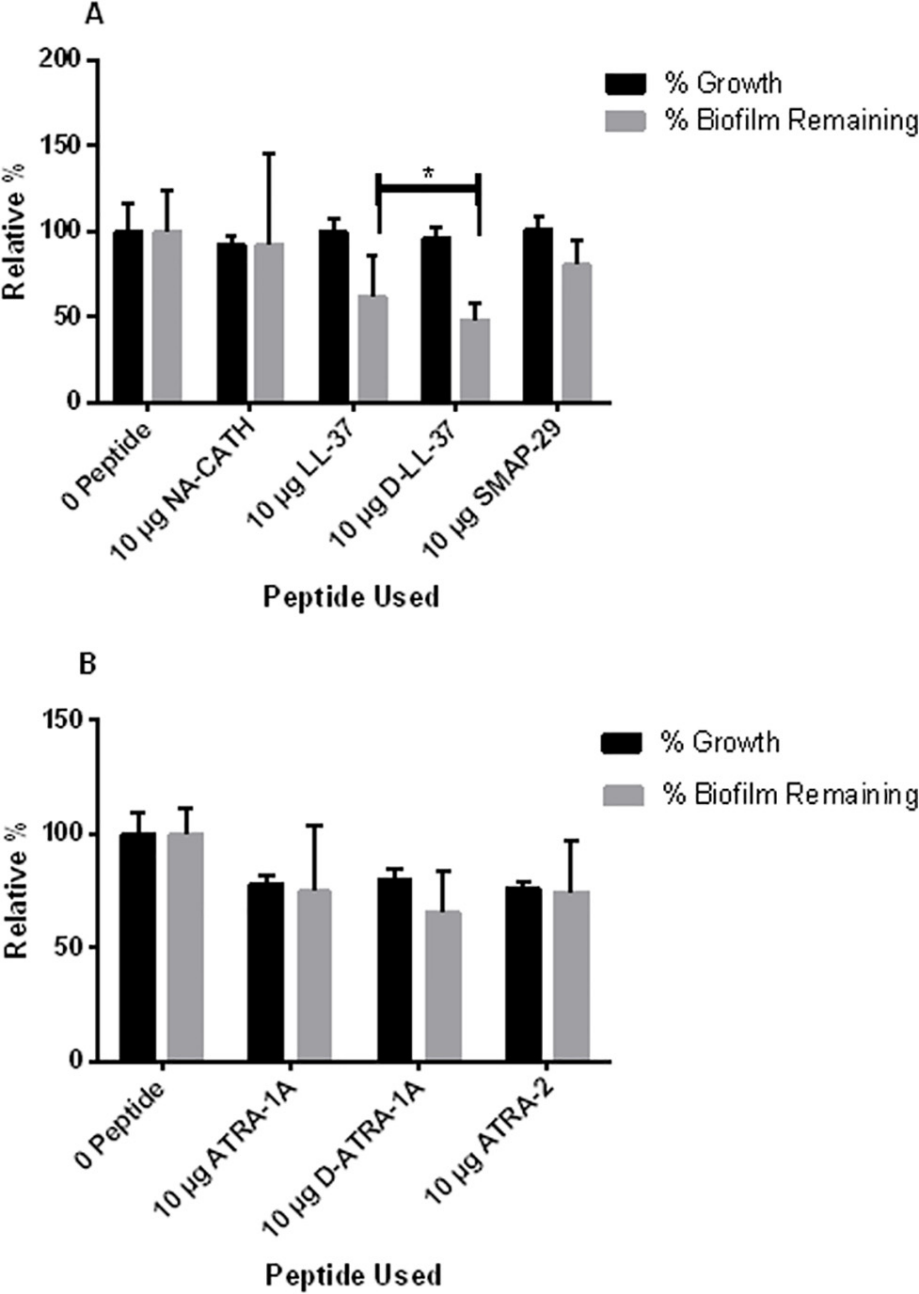
L- and D-LL-37 are able to disperse pre-formed *B*. *thailandensis* biofilms. A panel of cathelicidins were screened for the activity to disperse pre-formed biofilms. **A.** 10 μg cathelicidins NA-CATH (11.98 μM), LL-37 (11.13 μM), D-LL-37 (11.13 μM) and SMAP-29 (15.36 μM) and **B.** 10 μg NA-CATH derivatives ATRA-1A (35.19 μM), D-ATRA-1A (35.19 μM) and ATRA-2 (35.57) were added to 24h pre-formed biofilm and their growth and biofilm were measured via optical density after additional 24h incubation with peptides. Black bars indicate percent growth (absorbance at 600 nm) and grey bars represent the percent of the biofilm (absorbance at 590 nm) remaining after peptide treatment. Asterisk indicates student’s t test was performed to determine significance between LL-37 and D-LL-37 dispersion. The p-value for this was 0.196 leading to the conclusion that there was not a significant difference between biofilm dispersion of LL-37 and D-LL-37.

We have demonstrated that some of our cathelicidins can inhibit biofilm formation in *B thailandensis*. We wanted to know if these cathelicidin peptides could also disperse pre-formed biofilms. Therefore, a number of cathelicidins were chosen for the pre-formed biofilm dispersion assay as described in the methods. We demonstrated that LL-37 and D-LL-37 were able to disperse at least 50% of the pre-formed biofilm when 10 μg (11μM) peptide was added to 24h pre-formed biofilms (Fig 6), showing statistically equivalent activity. Other cathelicidins, SMAP-29 and NA-CATH (which demonstrated biofilm inhibition) did not demonstrate the ability to disperse pre-formed biofilms. Also, as expected, small NA-CATH derivative peptides (ATRA-1A, D-ATRA-1A, ATRA-2) showed no biofilm dispersion activity. The ability of D-LL-37 to disperse preformed *Burkholeria* biofilm has not been previously reported. These results suggest that LL-37 and D-LL-37 have a unique property that enables dispersion of preformed biofilm in this organism.

## Discussion

*B*. *thailandensis* and *B*. *pseudomallei* have a wide range of mechanisms for evading antibiotics and antimicrobial peptides. These mechanisms include, but are not limited to, having a more impermeable membrane, multi-drug-resistant efflux pumps, inactivation of host proteins, and modification of drug targets [10]. In this study, we demonstrate that certain peptides can evade these mechanisms and exhibit antimicrobial activity against *B*. *thailandensis*, despite its reported extreme peptide resistance. We also demonstrate that D-amino acid peptides exhibit comparable antimicrobial activity [15]. Finally, we describe the anti-biofilm activity of some of these peptides against *B*. *thailandensis*.

The modes of action of CAMPs against bacteria are varied and require further elucidation; however, two potentially co-existing mechanisms exist. The first model is that these CAMPs cause the formation of transmembrane pores, causing dissolution of the membrane potential and eventual destruction of the bacterial cell [47]. A second proposed mechanism includes the internalization of the CAMP which can then bind to internal targets and interfere with cell wall synthesis [48]. In humans, only one cathelicidin has been identified: LL-37. This cathelicidin is released by proteolysis from the C-terminus of CAP18 protein [49]. The LL-37 cathelicidin, as well as other cathelicidins with similar alpha-helical structures, has been demonstrated to associate with the bacterial membrane and cause bacterial death [50,51]. The effective killing concentration of these alpha-helical cathelicidin-type CAMPs has driven the search for new cathelicidins. Recently, a cathelicidin has been discovered in the species *Naja atra*, the Chinese cobra [25,52] which is effective against multiple bacteria [13–16]

Previous reports indicated that LL-37 is antimicrobial against *B*. *thailandensis* and *B*. *pseudomallei*. It has been reported that 15 μM LL-37 in 1 mM potassium phosphate buffer (PPB) kills 10^5^ cfu/mL of *B*. *thailandensis* [35]. Another study with *B*. *thailandensis* demonstrated that 5 strains of *B*. *thailandensis* were >90% killed at concentrations of 12.5 mg/L or greater in 1 mM PPB [33]. We obtained the same result, but in this study we have reported our results in terms of EC50 rather than lethal concentration. Research has also shown that LL-37 is effective at killing *B*. *pseudomallei*. One study reports that LL-37 effectively killed 24 strains of *B*. *pseudomallei* at a peptide concentration of 100 μM in 1 mM PPB [34], while another demonstrated >90% killing of 9 strains at concentrations of 6.25 mg/L (1.39 μM) or greater [33]. Under conditions of *B*. *pseudomallei* respiratory infection in mice, neutrophil granules were observed to be the predominant cell type seen in association with *B*. *pseudomallei* infection [39]. Since neutrophil granules are known to be a significant source of cathelicidins [40], our data and published results suggest a significant potential role of LL-37 expression during the infection of humans by *Burkholderia* species. We were able to demonstrate that LL-37, SMAP-29, NA-CATH, and small NA-CATH-derived ATRA peptides exert strong antimicrobial activity against *B*. *thailandensis*. Another group reported that the bovine cathelicidin BMAP-18 was antimicrobial against *B*. *pseudomallei* at 20 μM [53]. Together, these studies suggest that *B*. *thailandensis* and *B*. *pseudomallei* may be quite susceptible to cathelicidins as a class of peptides. A recent study also found that additional peptides, including the 12-aa peptide bactenecin, the hybrid peptide CA-MA, and RTA3, were antimicrobial against *B*. *pseudomalle*i [53], suggesting that there are peptides that appear to be effective against this organism, particularly those with predominantly helical properties.

In addition, we addressed the potential issue of proteolytic degradation of AMPs *in vivo* by bacterial proteases. Sieprawska-Lupa et al. demonstrated that mammalian hosts and numerous bacteria express proteases capable of degrading and inactivating LL-37 [54]. Therefore, we tested D-enantiomers, which we previously showed to be resistant to trypsin digestion [15], to compare their antimicrobial activity to that of the natural L-enantiomer. Our results show that for both LL-37 and ATRA-1A, the D-enantiomer exhibited antimicrobial activity comparable to that of the L-enantiomer, suggesting that bacterial proteases were not active against this peptide.

Our results also demonstrate that defensin peptides have at best a weak antimicrobial effect against *Burkholderia*. Human beta-defensins had previously been shown to be ineffective against *B*. *cepacia* or *B*. *pseudomallei* [18,43]. The alpha-defensins HNP-1 and HNP-2 both demonstrated poor antimicrobial performance against *B*. *thailandensis* in this work. For HNP-1, this is consistent with the literature on *B*. *pseudomallei* [55,56]. (No previous work was found on HNP-2’s effect on *Burkholderia pseudomallei*.) In addition, we tested HNP-3 and HNP-4 (Fig 4B) and found a similar lack of antimicrobial activity. This leads us to conclude that alpha-defensins in general do not exert strong antimicrobial activity against *B*. *thailandensis*. A fragment of hBD3 (Peptide 4 from hBD3) was also ineffective against *B*. *thailandensis*. Thus, *Burkholderia* does seem to be highly resistant to both classes of defensins.

It has also been demonstrated that both *B*. *thailandensis* and *B*. *pseudomallei* form biofilms *in vivo* [44,45], which may be a virulence factor [45]. We were able to demonstrate that the cathelicidins LL-37, D-LL-37, NA-CATH, and SMAP-29 are capable of biofilm inhibition in *B*. *thailandensis* each at similar extents of ~50% inhibition at 3 μg/ml. This is in agreement with the published capability of LL-37 to inhibit biofilm formation in *Pseudomonas* [46]. In addition, we demonstrated the new result of the ability of D-LL-37 to disperse pre-formed biofilms. Thus, the biofilm inhibition we demonstrate in this work may be a crucial component of the activity of cathelicidin-derived peptides as possible therapeutics.

Novel approaches to treatment for *Burkholderia* infections are critically needed, especially for treatment of melioidosis. A novel peptide-based treatment for melioidosis would ideally include both antimicrobial activity and biofilm inhibition, and may take the form of a topical application. In this work, we have demonstrated the effects of LL-37, SMAP-29, and NA-CATH as both antimicrobial and anti-biofilm peptides, and showed promising results of short, synthetic peptides, such as ATRA1. We have also extended previous studies [35] showing here that an all-D-enantiomer of LL-37, which is resistant to proteolytic degradation, maintains antimicrobial activity as well as significant anti-biofilm properties against *B*. *thailandesnsis*. The results of this study illustrating the susceptibility of *B*. *thailandensis* to cathelicidin-like peptide killing, resistance to defensins, and the ability of D- and L-LL-37 peptides to inhibit biofilm formation may provide a new understanding of the potential use for peptides, perhaps as topical applications, in melioidosis infection.

## Supporting Information

S1 Fig Biofilm activity of NA-CATH imperfect repeat derivatives against *B*. *thailandensis*. Minimal biofilm inhibition is demonstrated for **B.** D-ATRA-1A, while not biofilm inhibition is demonstrated for **A.** ATRA-1A, **C.** ATRA-1, and **D.** ATRA-2. Growth (absorbance at 600 nm) is shown by black bars; growth with no peptide was set to 100%. Biofilm (gray bars) was detected on a polystyrene 96-well plate at 37°C after 48 h of growth in MVBM and detected as absorbance of crystal violet stain (590 nm). Each experiment is representative of 3 individual experiments.
(TIF)
