# Supplementary figures and images for: Snake Cathelicidin NA-CATH and Smaller Helical Antimicrobial Peptides Are Effective against Burkholderia thailandensis
Source: PLoS Negl Trop Dis. 2015 Jul 21;9(7):e0003862. doi: 10.1371/journal.pntd.0003862 (PMC4510350; doi:10.1371/journal.pntd.0003862)

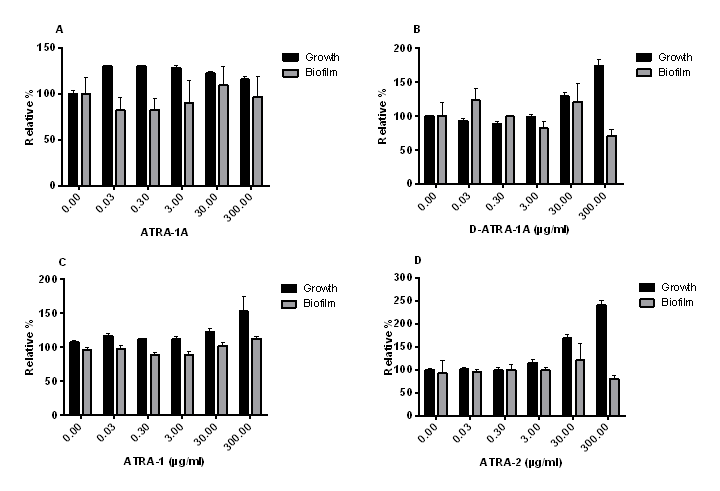

Supplement: S1 Fig — Minimal biofilm inhibition is demonstrated for B. D-ATRA-1A, while not biofilm inhibition is demonstrated for A. ATRA-1A, C. ATRA-1, and D. ATRA-2. Growth (absorbance at 600 nm) is shown by black bars; growth with no peptide was set to 100%. Biofilm (gray bars) was detected on a polystyrene 96-well plate at 37°C after 48 h of growth in MVBM and detected as absorbance of crystal violet stain (590 nm). Each experiment is representative of 3 individual experiments. (TIF) [file pntd.0003862.s001.tif]
